# Social and Behavior Change Communication Interventions Delivered Face-to-Face and by a Mobile Phone to Strengthen Vaccination Uptake and Improve Child Health in Rural India: Randomized Pilot Study

**DOI:** 10.2196/20356

**Published:** 2020-09-21

**Authors:** Mira Johri, Dinesh Chandra, Karna Georges Kone, Marie-Pierre Sylvestre, Alok K Mathur, Sam Harper, Arijit Nandi

**Affiliations:** 1 Centre de Recherche du Centre Hospitalier de l’Université de Montréal (CRCHUM) Montréal, QC Canada; 2 Département de gestion, d’évaluation, et de politique de santé École de santé publique de l'Université de Montréal Montréal, QC Canada; 3 Independent consultant New Delhi India; 4 Principal Technical Advisor for Health Financing at Management Sciences for Health (MSH)/USAID Port-au-Prince Haiti; 5 Département de médecine sociale et préventive École de santé publique de l'Université de Montréal Montréal, QC Canada; 6 Indian Institute of Health Management Research University Jaipur India; 7 Department of Epidemiology, Biostatistics & Occupational Health McGill University Montreal, QC Canada; 8 Department of Public Health Erasmus Medical Centre Rotterdam Netherlands; 9 The Institute for Health and Social Policy McGill University Montreal, QC Canada

**Keywords:** randomized controlled trial, immunization programs, child health, mHealth, health promotion, health services accessibility, implementation science, pilot projects, developing countries, global health

## Abstract

**Background:**

In resource-poor settings, lack of awareness and low demand for services constitute important barriers to expanding the coverage of effective interventions. In India, childhood immunization is a priority health strategy with suboptimal uptake.

**Objective:**

To assess study feasibility and key implementation outcomes for the Tika Vaani model, a new approach to educate and empower beneficiaries to improve immunization and child health.

**Methods:**

A cluster-randomized pilot trial with a 1:1 allocation ratio was conducted in rural Uttar Pradesh, India, from January to September 2018. Villages were randomly assigned to either the intervention or control group. In each participating village, surveyors conducted a complete enumeration to identify eligible households and requested participation before randomization. Interventions were designed through formative research using a social marketing approach and delivered over 3 months using strategies adapted to disadvantaged populations: (1) mobile health (mHealth): entertaining educational audio capsules (edutainment) and voice immunization reminders via mobile phone and (2) face-to-face: community mobilization activities, including 3 small group meetings offered to each participant. The control group received usual services. The main outcomes were prespecified criteria for feasibility of the main study (recruitment, randomization, retention, contamination, and adoption). Secondary endpoints tested equity of coverage and changes in intermediate outcomes. Statistical methods included descriptive statistics to assess feasibility, penalized logistic regression and ordered logistic regression to assess coverage, and generalized estimating equation models to assess changes in intermediate outcomes.

**Results:**

All villages consented to participate. Gaps in administrative data hampered recruitment; 14.0% (79/565) of recorded households were nonresident. Only 1.4% (8/565) of households did not consent. A total of 387 households (184 intervention and 203 control) with children aged 0 to 12 months in 26 villages (13 intervention and 13 control) were included and randomized. The end line survey occurred during the flood season; 17.6% (68/387) of the households were absent. Contamination was less than 1%. Participation in one or more interventions was 94.0% (173/184), 78.3% (144/184) for the face-to-face strategy, and 67.4% (124/184) for the mHealth strategy. Determinants including place of residence, mobile phone access, education, and female empowerment shaped intervention use; factors operated differently for face-to-face and mHealth strategies. For 11 of 13 intermediate outcomes, regression results showed significantly higher basic health knowledge among the intervention group, supporting hypothesized causal mechanisms.

**Conclusions:**

A future trial of a new intervention model is feasible. The interventions could strengthen the delivery of immunization and universal primary health care. Social and behavior change communication via mobile phones proved viable and contributed to standardization and scalability. Face-to-face interactions remain necessary to achieve equity and reach, suggesting the need for ongoing health system strengthening to accompany the introduction of communication technologies.

**Trial Registration:**

International Standard Randomized Controlled Trial Number (ISRCTN) 44840759; https://doi.org/10.1186/ISRCTN44840759

## Introduction

### Background and Rationale

Expanding coverage of effective interventions is a critical challenge for many low- and middle-income countries (LMICs). In addition to technical improvements in service delivery, improving coverage often hinges critically on enhanced awareness and demand for services on the part of beneficiaries. Furthermore, in settings of low literacy, deep poverty, and poor access to information, behavior change is extremely challenging.

Immunization is a priority health strategy for LMIC policy makers seeking to advance the 2030 United Nations (UN) Sustainable Development Goals (SDGs) due to its inherent value in reducing the burden of disease and its potential role as a lever for health system strengthening. Immunization reaches more households than any other health service, bringing communities into regular contact with the health system [1]. The immunization platform can potentially be used to strengthen the delivery of universal primary health care, universal health coverage, and meet other SDG targets [1]. This approach may be particularly salient in areas where vaccination delivery systems function reliably, but important gaps exist in the delivery of other health services. In these contexts, increasing immunization coverage offers a potential pathway to expand the range and reach of health services and to advance a holistic health agenda.

In India, the government has prioritized immunization, making remarkable gains in recent years. However, coverage continues to fall short of the target to fully immunize 90% of India’s infants against 7 vaccine-preventable diseases by 2020 [2]. Nevertheless, immunization delivery now outperforms other services, offering a potential lever for system strengthening. In rural north India, for example, research shows that high-priority primary care interventions, including vaccination, are being delivered quite well, whereas other basic health promotion and prevention services are largely not provided, constituting a critical missed opportunity for population health [3]. On the basis of analysis of Indian immunization program data, achieving and sustaining vaccine coverage targets especially requires new strategies to address gaps in beneficiary demand [2]. Recent systematic reviews and meta-analyses have demonstrated that knowledge translation and education strategies, such as those offering education at village meetings or at home, are likely to improve vaccination coverage [4,5]. However, strategies based on face-to-face communication may be challenging to standardize and deliver at scale.

The widespread availability of mobile phones in LMICs has stimulated interest in the potential of mobile health (mHealth) interventions to achieve health objectives. A recent systematic review found that mHealth interventions can improve maternal and neonatal service delivery and that text-based vaccination reminders are associated with improved vaccination coverage [6]. Although their potential for scalability at low cost is attractive, whether mHealth interventions can be effective for highly disadvantaged populations facing substantial barriers due to poverty, low literacy, and gender norms is uncertain.

### Goal of This Study

We developed the *Tika Vaani* (*vaccine voice* in Hindi) model to educate beneficiaries about immunization and basic child health themes, dispel misinformation, and empower households to better care for their children and themselves. A key distinguishing feature of the model is that it integrates an mHealth component to increase standardization and scalability of social and behavior change communication. The interventions were delivered through small face-to-face meetings and via mobile phone using strategies (context-appropriate audio messages delivered via automated phone calls) adapted to disadvantaged populations. We conducted a pilot randomized controlled trial (RCT) applying an implementation research lens to inform a future large-scale RCT. This paper presents the main (quantitative) evaluation of the pilot trial focusing on two objectives: (1) to assess the feasibility of processes critical to the success of the main study (recruitment, randomization, retention, and contamination) and intervention uptake (adoption) and (2) to study key implementation outcomes to optimize successful delivery of the interventions at scale [7]. Objectives pertained to cluster and individual levels. A companion paper presents findings related to intervention fidelity, acceptability, and appropriateness using mixed (qualitative and quantitative) methods (Pérez et al, unpublished data, 2020).

## Methods

### Trial Design

In keeping with the plan for the main study, this pilot study adopted a cluster-randomized design with a 1:1 allocation ratio. A cluster design was chosen owing to the nature of the study interventions: face-to-face interventions are structured around communities rather than individuals, whereas mHealth interventions have a possibility of contamination. Clusters were rural villages in a district of Uttar Pradesh (UP), India. Villages were randomly assigned to either the intervention or control group (CG). The protocol was registered in a WHO International Clinical Trials Registry Platform-compliant registry (ISRCTN44840759 doi.org/10.1186/ISRCTN44840759). There were no important changes to methods after trial commencement. We originally sought to register the trial in the Clinical Trials Registry–India (CTRI), which is free of charge and has as the mission to enroll all clinical trials conducted in India. The CTRI took several months to follow-up; in the interim, we applied to a different registry. Owing to the delay caused by waiting and changing registries, the trial was registered shortly after patient enrollment was completed.

### Participants

#### Setting and Location

India’s most populous state of over 200 million residents, UP is an area of national focus due to lagging health and development indicators. Hardoi (population 4 million; under-5 mortality rate 118 per 1000; cf. UP under-5 mortality rate 90 per 1000, India under-5 mortality rate 57.3 per 1000) [8,9]) is a low-performing, rural district within UP comprising 19 administrative blocks. Thanks to recent Government of India (GoI) initiatives, the proportion of fully immunized children aged between 12 and 23 months in Hardoi district rose from 26.5% in 2007-2008 [10] to an estimated 65.9% (95% CI 62.0%-69.8%) in 2018 when this study was conducted [2]. A single administrative block of the Hardoi district was selected for this pilot based on criteria reflecting logistics and needs.

#### Eligibility Criteria

Villages (clusters) were eligible for inclusion if they had less than 4000 inhabitants and were located in Bawan Block, Hardoi, UP. In participating villages, interventions were offered to all residents. Participants in the baseline survey were consenting primary caregivers of children aged between 0 and 12 months residing in a study village. We excluded those who were not able to understand and speak Hindi or Urdu or those who did not intend to reside in the village for the study duration (6 months). The same individuals were approached for the end line survey.

The survey sampling unit was the household. We conducted a door-to-door census of the village and cross-checked administrative records from the Anganwadi workers (AWW) and Accredited Social Health Activists (ASHA) to identify all households containing a child in the age range of 0 to 12 months within each village. These households constituted our primary target group.

### Interventions

#### Formative Research

From January 1, 2017, to January 10, 2018, we conducted formative research using a social marketing approach to inform intervention design [11]. An iterative, participatory approach involving cocreation was favored to validate the need for the interventions, to make the interventions more compelling and linguistically and culturally appropriate, and to tailor approaches to different user segments [11]. Content was designed by Gram Vaani, an Indian social enterprise specializing in community media platforms for low-literacy rural populations, and Jagriti, a local NGO. Content fostered equity, empowerment, and social inclusion through positive portrayals of diverse characters. Technical experts assured information quality, including members of the research team and India’s Ministry of Health and Family Welfare [11]. Extensive adaptations to interventions were made to meet target population needs during the formative research phase [12]. During the pilot RCT, all intervention components were frozen for evaluation, and deviations to intervention fidelity were systematically monitored (Pérez et al, unpublished data, 2020.

#### Pilot RCT

The study interventions took place over a 3-month period and offered social and behavior change communication (SBCC) for members of the general public in rural Indian villages, addressing topics related to child health. The primary target group was families residing in a selected village with a child in the age range of 0 to 12 months. Although vaccination was the primary focus of the study, the SBCC interventions addressed additional areas stipulated to be co-delivered with immunization during Village Health and Nutrition Days (VHNDs), such as health education related to health care entitlements; prevention, recognition, and management of common infectious diseases (diarrhea, pneumonia, dengue, and chikungunya); nutrition; and water, sanitation, and hygiene (WASH).

SBCC materials were delivered through 2 channels: (1) mHealth: educational audio capsules in entertaining formats (edutainment) and voice reminders for immunization sessions broadcast via mobile phone and (2) face-to-face: community mobilization activities, consisting of 1 large introductory meeting to introduce the project to communities and 3 small meetings offered to each participant covering specific themes. For the mHealth component, push messages (automated dial outs) and voice-based reminders were privileged owing to low education level and comfort with technology. For the face-to-face component, small group meetings were held separately for men and women and in different geographical locations within villages to ensure ease of communication. mHealth vaccination reminders were based on the child’s birthdate and offered only to the target group; however, other interventions (mHealth edutainment and face-to-face meetings) were open to all village residents. Community workers (AWW and ASHAs) were encouraged to participate and received advance access to intervention materials. All interventions were free of charge to end users. The CG received standard GoI health and welfare services.

The mHealth strategy (Tika Vaani SBCC Version 1.5, released on July 7, 2017) was designed and delivered by Gram Vaani, a social tech startup incubated out of the Indian Institute of Technology Delhi, using the Mobile Vaani Interactive Voice Response System. Access was free and open to anyone who called the number. The participants could give a missed call to access the platform, and as a result receive a callback enabling them to access all content, record any queries or feedback, or be connected to a live expert. To simplify access, consenting households in intervention villages with children aged less than 12 months at baseline received automated outbound calls twice per week. In total, 26 content pieces were offered. In addition, child vaccination reminders were sent to target group households. Small group meetings lasting approximately 1 hour involving 2 trained facilitators with a minimum of 12 years of education were held once per month and open to all village residents. The access number was shared at each meeting. A video describing Mobile Vaani is available [13]. Additional information relevant to scale up and replication of the Tika Vaani system is available [11] and content is accessible [14]. A comprehensive intervention description is provided in Multimedia Appendix 1 [15]. The evaluation was conducted by a team of academic specialists distinct from the developers.

### Outcomes

The pilot study considered a range of implementation outcomes (Table 1) [16].

**Table 1:** Outcome variables and data sources for the Tika Vaani social and behavior change communication pilot study.

Outcomes		Definition	Approach	Analysis sample	Data sources
**Primary outcomes^a^**					
	Feasibility of the future main study	Ex-ante success criteria Recruitment Randomization Retention Contamination	Quant^b^	IG^c^ and CG^d^	Project records (all) IVR^e^ platform HH^f^ surveys (contamination)
	Uptake (adoption)	Participation in Small group meetings mHealth^g^	Quant	IG	Project records (meetings) IVR platform (mHealth)
**Secondary outcomes**					
	Acceptability and appropriateness	Perception among stakeholders that an intervention is agreeable, suitable, relevant, useful, and credible	Mixed methods^h^	IG	Refer to the study by Pérez et al (unpublished data, 2020)
	Fidelity	Ability to deliver the interventions as planned	Mixed methods	IG (and CG)	Refer to the study by Pérez et al (unpublished data, 2020)
	Coverage	The degree to which a population eligible to benefit from an intervention actually receives it	Quant	IG	HH surveys Project records (meetings) IVR platform (mHealth)
	Adequacy of the program theory	Intermediate outcomes reflecting changes in knowledge, attitudes, and practices of end users	Quant	IG and CG	HH surveys

^a^Outcomes and definitions adapted from the study by Peters et al [16].

^b^Quant: quantitative.

^c^IG: intervention group.

^d^CG: control group.

^e^IVR: interactive voice response.

^f^HH survey: household survey.

^g^mHealth: mobile health.

^h^Mixed methods: quantitative and qualitative.

#### Primary Outcomes

We established ex-ante criteria for the feasibility of the main study related to recruitment, randomization, retention, and contamination. We were concerned about contamination among treatment groups for mHealth services, as the phone number is easily shared. We viewed a contamination proportion exceeding 15% as a threat to the feasibility of adopting a cluster-randomized design with village as the unit of randomization and geographical distances between villages (mean 15 km; range 5 km-50 km) similar to those in the pilot. As health interventions must achieve sufficient uptake to impact population health, we also established minimum criteria for participation in the new interventions.

#### Secondary Outcomes

**Figure 1:**
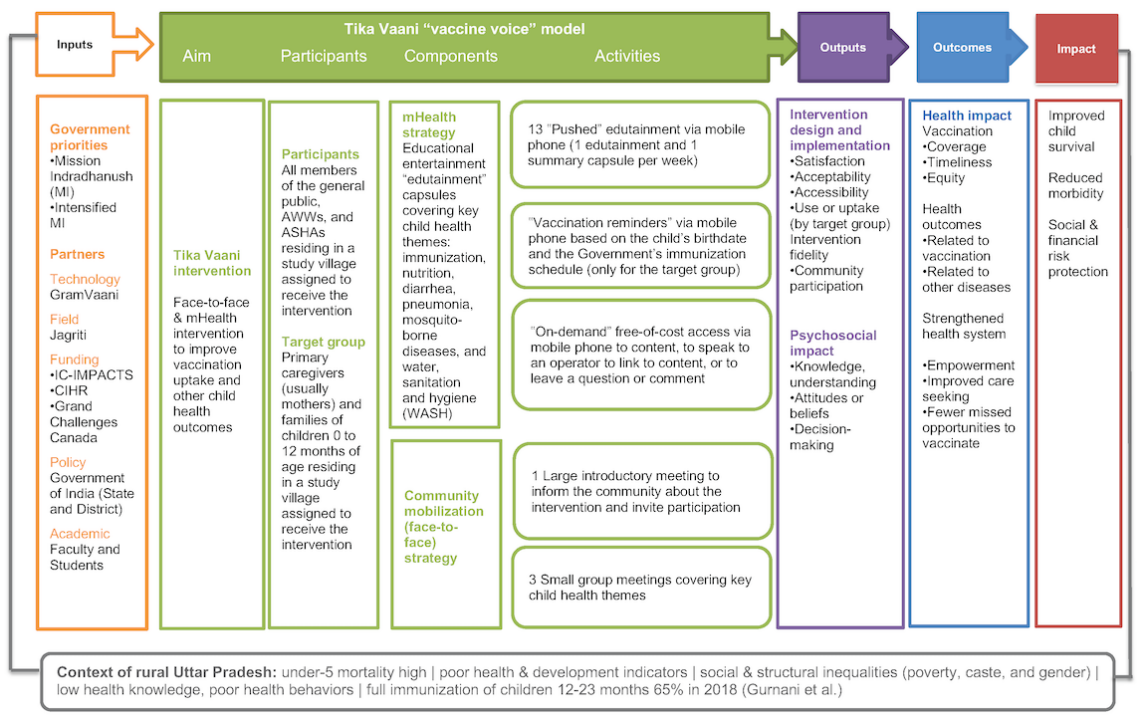
Conceptual model of the intervention. ASHAs: Accredited Social Health Activists; AWWs: Anganwadi Workers; CIHR: Canadian Institutes for Health Research; IC-IMPACTS: the India-Canada Centre for Innovative Multidisciplinary Partnerships to Accelerate Community Transformation and Sustainability; WASH: water, sanitation, and hygiene.

We present quantitative findings for 2 secondary outcomes: (1) coverage or the extent to which the interventions reached specific populations and (2) adequacy of the program theory. We constructed a logic model describing the hypothesized program impact pathway and mechanisms of action (Figure 1) and adapted an established vaccination communication taxonomy to define indicators [17]. We compared treatment groups on *outputs* (intermediate outcomes, such as knowledge and attitudes) related to the intervention theory of change.

### Data Sources

Quantitative data were collected from the following sources:

- Face-to-face surveys: Interviewer-administered household surveys were conducted in all participating study villages. Surveys were administered at baseline before random group assignment and approximately 5 months later following interventions at end line.
- Project administrative records: Standardized forms to assess delivery of study procedures and interventions were maintained by field staff to facilitate structured observation and data capture.
- Interactive voice response (IVR) system: The IVR system automatically recorded all calls to the platform. IVR data can be disaggregated by various fields including caller phone number, date, time, frequency, duration (seconds), call type, content type, and user characteristics. IVR data were linked to phone numbers provided by target households during the baseline survey to map calls sent and/or received.

### Variables

We measured the use of the IVR through the number and duration of calls from a unique phone number. We considered that an mHealth item was *received* if the caller remained connected for 80% or more of the item duration. Contamination was defined as the proportion of calls originating from control villages and assessed using 2 data sources: (1) the IVR system to identify unknown numbers and trace nonregistered callers to identify call origin and (2) questions were included about intervention use in the end line survey administered to the treatment and CGs (Multimedia Appendix 1). To construct wealth quintiles, we performed principal component analysis to create a relative index of household wealth from a list of assets used in major household surveys [10,18]. We used this index to divide the sample into quintiles [19]. A similar approach was used to construct women’s empowerment terciles. Caste was represented in 4 categories (general, other backward caste, scheduled caste, or scheduled tribe) ranging from most to least advantaged, as for Indian national surveys. By convention, caste categories are applied to all population groups, irrespective of religion. For intervention group (IG) households only, implementation teams assessed whether households were able to attend small group meetings based on geographical distance from their place of residence to anticipated meeting sites. This categorization was made at the study baseline, before undertaking interventions.

### Sample Size

Although the pilot was a cluster-randomized two-group study, the study size was based on the rate of contamination among controls. We estimated the required sample size needed for the CG using methods for a one-group descriptive study. We assumed that the true proportion of contamination (calls originating from controls) was 10%, that contamination was most likely to arise from parents of young children, and that there would be 20 households with children aged less than 12 months per village. On the basis of these inputs and using a binomial (*exact*) calculation, we would require 200 households in the CG to detect a 95% confidence interval of 6.2% to 15.0% [20]. The total sample size for the pilot was therefore set at double this number or 400 households.

### Village Selection

The sampling frame was informed by the 2011 census [21], which indicated that the Bawan Block had a total population of approximately 234,000, including 217,000 rural residents distributed among 143 villages. We eliminated 3 urban villages, 11 villages with a population exceeding 4000, 15 villages recorded as having 0 population, and 57 villages in which we had previously worked (so as to gain experience in a treatment-naïve population). This left a sampling frame of 57 villages, from which an initial 20 villages were randomly selected using Microsoft Excel. The number of children in the target age range per village was not known in advance of the baseline survey. As villages vary in size, to attain our sample size target, we decided a priori that (1) any village with more than 1 but less than 10 children in the target age range would be retained and another randomly selected village would also be added and (2) villages with no children in the target age range would be dropped.

### Randomization

#### Sequence Generation and Allocation Concealment

Villages were assigned to either intervention or control using simple randomization with a 1:1 allocation following a computer-generated randomization schedule. The random allocation sequence was generated at the Centre de recherche du Centre hospitalier de l’Université de Montréal by a professional statistician (MPS) using commands for random samples and permutations in the R environment for statistical computing [22] and kept in a password-protected computer. The statistician was not involved in study implementation. Before the release of the randomization code, only the statistician had access to the allocation sequence. Randomization code was released all at once, and treatment groups were assigned only after completing all recruitment procedures and baseline measurements.

#### Implementation

Field team leaders enrolled clusters by contacting village officials in person to explain study aims and activities and request consent to participate. Subsequently, in each participating village, surveyors conducted a complete enumeration to identify all households with children in the target age range and directly approached all such households to request participation in the baseline survey and pilot study. Consent was sought before randomization. No advertisements were used for recruitment, and no incentives or rewards were offered for participation. Surveyors communicated group assignments personally to households.

#### Blinding

Due to the nature of the interventions, neither participants nor those delivering interventions were blinded to the group assignment. We hired independent surveyors at end line to assess study outcomes. These surveyors were not informed about study aims or group assignments.

### Statistical Methods

#### Descriptive Analyses

We used counts, frequencies, and proportions to summarize categorical data, and means and standard deviations for continuous variables. We assessed bivariate associations using univariable logistic regression for continuous variables and the chi-square test for categorical variables.

#### Multivariable Analyses

#### Coverage

We studied the degree to which target beneficiaries (IG households with a child aged less than 12 months at baseline) received the interventions.

To investigate patterns of uptake, we developed separate models for each intervention component: immunization reminders (mHealth), edutainment capsules (mHealth), and small group meetings (face-to-face). Outcomes were modeled as binary (0 uptake vs 1 or more instances of uptake). We followed guidance for the use of logistic regression in small data sets [23,24]. To identify candidate predictors of uptake and use, we developed a conceptual framework informed by the scientific literature and expert knowledge (Multimedia Appendix 1). The conceptual framework considered socioeconomic determinants, physical and infrastructure barriers, access barriers related to mobile phone use within households, and women’s empowerment. Together, these variables represented 17 degrees of freedom. To develop the full models for implementation, we empirically refined the choice of variables to respect a minimum of roughly 10 events per variable for accurate prediction of binary outcomes [23]. Specifically, we excluded candidate variables if the bivariate chi-square test showed no relationship between predictor and outcome at the level of *P*<.25. We fit full statistical models using the Firth (penalized maximum likelihood) logistic regression to avoid overfitting [23] and handle data separation. The final models were fit within a cluster bootstrap algorithm (1000 iterations).

To study the determinants of intensity of participation, we repeated analyses specifying ordered logistic regression models for 2 outcomes: (1) the number of mHealth items heard and (2) the number of small group meetings attended. We tested the proportional odds assumption using an approximate likelihood ratio test [25]. All models used robust standard errors to account for clustering.

#### Adequacy of the Program Theory

We studied intervention impact on intermediate outcomes using generalized estimating equations (GEE): (1) we used the differences-in-differences method to study changes in variables measuring immunization knowledge in the 2 study groups between the baseline survey and the end line survey using unadjusted regression coefficients (with their 95% CIs) for the interaction between group (intervention or control) and time period (end line or baseline) [26]. (2) To assess knowledge of other basic health topics (assessed only at study end line), we estimated the probability of correct responses at the end line among those receiving the intervention (vs controls). All GEE models estimated binary outcomes with an exchangeable correlation structure adjusted for village-level clustering and robust standard errors. We ran crude models and models adjusted for unbalanced variables following randomization and prespecified potential confounders.

Feasibility outcomes were assessed using the intention-to-treat (ITT) sample; no clusters and no participants were excluded. Analyses of intervention uptake and coverage used the ITT IG; no clusters and no participants randomized to the IG were excluded. To assess the program theory, we analyzed intermediate outcomes using the sample that participated at both baseline and end line, for which 0 clusters, 68 households, and 69 caregivers were lost to follow-up, which is equivalent to an observational sample. For 2 households, missing data on caste were imputed based on the locality of residence within the village. There were no other missing data. Analyses were conducted in Stata 15 (Stata Corporation).

### Research Ethics and Informed Consent

Permission was granted by the Institutional Committee for Ethics and Review of Research, Indian Institute of Health Management Research, Jaipur, on January 10, 2017, and by the Comité d’éthique de la recherche du Centre hospitalier de l’Université de Montréal (Research Ethics Committee of the University of Montreal Hospital) on January 11, 2017 (Reference number 16.084). All participants provided written, in-person informed consent. After completing the study, we offered CG residents access to the mHealth interventions.

## Results

### Participants

**Figure 2:**
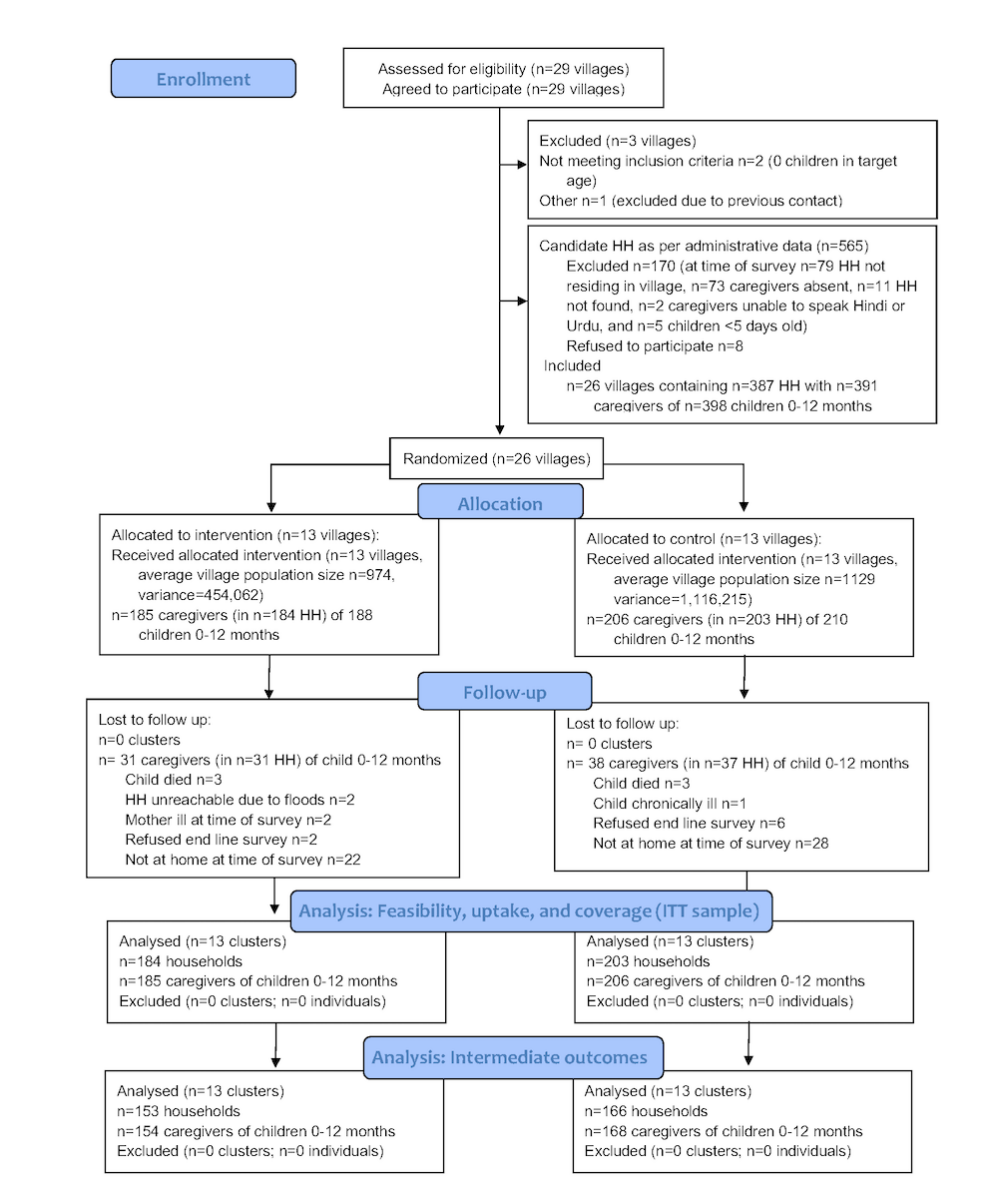
Flow diagram of the parallel group cluster trial. ITT: intention-to-treat.

The baseline survey and recruitment took place from January 19 to February 19, 2018. We approached 29 villages and 100% (29/29) consented to participate. Recruitment of individual participants was complicated by gaps in administrative data, as 13.9% (79/565) of recorded households were in fact nonresident. Only 1.4% (8/565) of the candidate households did not consent to participate. A total of 391 (185 *IG* and 206 *CG*) caregivers of children aged 0 to 12 months in 387 (184 IG and 203 CG) households in 26 (13 IG and 13 CG) villages were included in the study (Figure 2). Interventions were delivered from March 21 to July 9, 2018. The end line survey took place from July 17 to August 20, 2018, during the annual monsoon floods. Many households (17.6%, 68/387) were absent during the study end line; loss to follow-up was non-differential (31 IG and 37 CG). The trial ended when planned activities were successfully completed; Figure 2 describes the progress of participants through the trial.

Characteristics of the participating individuals (Table 2) and villages (Table 3) were similar across treatment groups at baseline. The CG was advantaged in terms of assets (wealth quintiles) and cell phone network quality.

**Table 2:** Baseline characteristics of participating households, by treatment group.

Variable^a^		Intervention (n=184), n (%)	Control (n=203), n (%)	All participants (n=387), n (%)
**Wealth index (quintile)**				
	(Q1) Lowest	48 (26.1)	30 (14.8)	78 (20.2)
	(Q2)	37 (20.1)	40 (19.7)	77 (19.9)
	(Q3)	37 (20.1)	41 (20.2)	78 (20.2)
	(Q4)	28 (15.2)	49 (24.1)	77 (19.9)
	(Q5) Highest	34 (18.5)	43 (21.2)	77 (19.9)
**Religion^b^**				
	Hindu	181 (98.4)	176 (86.7)	357 (92.3)
	Muslim	3 (1.6)	27 (13.3)	30 (7.8)
**Caste^c^**				
	General	38 (20.7)	37 (18.2)	75 (19.4)
	Other backward caste	89 (48.4)	80 (39.4)	169 (43.7)
	Scheduled caste	57 (31.0)	86 (42.4)	143 (37.0)
**Maternal education (years)**				
	None (0)	62 (33.7)	75 (37.0)	137 (35.4)
	Primary (1-8)	85 (46.2)	84 (41.4)	169 (43.7)
	Secondary (9-12) or more	37 (20.1)	44 (21.7)	81 (20.9)
**Paternal education (years)**				
	None (0)	29 (15.8)	26 (12.8)	55 (14.2)
	Primary (1-8)	90 (48.9)	99 (48.8)	189 (48.8)
	Secondary (9-12) or more	65 (35.3)	78 (38.4)	143 (37.0)
**HH lives far from meetings^d^**				
	No	142 (77.2)	N/A^e^	N/A
	Yes	42 (22.8)	N/A	N/A
**Cell phone network poor**				
	No	163 (88.6)	203 (100.0)	366 (94.6)
	Yes	21 (11.4)	0 (0.0)	21 (5.4)
**HH owns a mobile phone**				
	No	11 (6.0)	15 (7.4)	26 (6.7)
	Yes	173 (94.0)	188 (92.6)	361 (93.3)
**In this HH, mother has phone**				
	No	106 (57.6)	134 (66.0)	240 (62.0)
	Yes	78 (42.4)	69 (34.0)	147 (38.0)
**In this HH, father has phone**				
	No	68 (37.0)	76 (37.4)	144 (37.2)
	Yes	116 (63.0)	127 (62.6)	243 (62.8)
**In this HH, someone else has phone**				
	No	162 (88.0)	166 (81.8)	328 (84.8)
	Yes	22 (12.0)	37 (18.2)	59 (15.3)
**Mother’s phone access**				
	Cannot access	90 (48.9)	88 (43.4)	179 (45.0)
	Can use easily	94 (51.1)	115 (56.7)	209 (54.0)
**Mother can dial phone**				
	No	47 (25.5)	55 (271)	102 (26.4)
	Yes	137 (745)	148 (72.9)	285 (73.6)
**Mother’s frequency of phone use**				
	Rarely	27 (14.7)	30 (14.8)	57 (14.7)
	When needed	102 (55.4)	106 (52.2)	208 (53.8)
	Almost daily	55 (29.9)	67 (33.0)	122 (31.5)
**Women’s empowerment score (tercile)**				
	Lowest	83 (45.1)	94 (46.3)	177 (45.7)
	Average	74 (40.2)	84 (41.4)	158 (40.8)
	Highest	27 (14.7)	25 (12.3)	52 (13.4)
**Permission to attend village meetings**				
	No	111 (60.3)	107 (52.7)	218 (56.3)
	Yes	73 (39.7)	96 (47.3)	169 (43.7)

^a^Baseline data are presented for the intention-to-treat sample of 387 households (184 IG and 203 CG).

^b^This is the religion of the household head.

^c^Caste categories from most to least advantaged: general, other backward caste, and scheduled caste. The scheduled tribe category is missing, as there are no tribes in the study area.

^d^HH: household.

^e^N/A: not applicable.

**Table 3:** Baseline characteristics of participating villages, by treatment group.

Variable^a^		Intervention	Control	All
**Village population, mean (SD)^b^**				
	Total	974 (673.84)	1129 (1056.51)	1051 (871.79)
	Total 0-6 years	166 (111.05)	188 (171.36)	177 (141.95)
	Total SC^c^	225 (194.85)	422 (484.76)	323 (375.62)
**Village electrification, n(%)**				
	No electricity	1 (7.7)	2 (15.4)	3 (11.5)
	Less than 6 hours	2 (15.4)	0 (0.0)	2 (7.7)
	More than 6 hours	10 (76.9)	11 (84.6)	21 (80.8)
**Characteristics of participating HH^d^, mean (SD)**				
	Number of Muslim HH per village	31 (61.39)	98 (168.96)	65 (129.25)
	Number of eligible HH^e^ per village	14 (7.39)	16 (12.46)	15 (10.07)
	% poor^f^ (Q1+Q2) per village	44.6 (0.19)	34.8 (0.23)	39.7 (0.21)
	% better off (Q4+Q5) per village	39.3 (0.26)	47.5 (0.28)	43.4 (0.27)
	% SC per village	31.0 (0.31)	41.5 (0.34)	36.3 (0.32)
	% of mothers with 0 schooling per village	32.4 (0.17)	41.0 (0.24)	36.7 (0.21)
	% of fathers with 0 schooling per village	14.6 (0.12)	9.8 (0.09)	12.2 (0.11)

^a^Baseline data are presented for the intention-to-treat sample of 26 villages (13 IG and 13 CG) containing 387 households (184 IG and 203 CG).

^b^Data from the 2011 Census of India.

^c^SC: scheduled caste (least privileged).

^d^HH: household.

^e^Eligible household: at least one child aged less than 12 months at baseline.

^f^Poor versus better off households based on asset indices (wealth quintiles).

### Primary Outcomes

Ex-ante criteria were fully satisfied (Table 4). Results from 2 independent sources demonstrated a very low (1% or less) rate of contamination (Table 4; Multimedia Appendix 1). Uptake of interventions (adoption) was very high; overall participation in one or more new interventions was 94.0% (173/184), 78.3% (144/184) for the face-to-face channel, and 67.4% (124/184) for the mHealth channel (Table 4). A total of 38.0% (70/184) of participating households used the mHealth intervention weekly. Together, these results confirm the feasibility of the future main study and demonstrate the potential to impact population health.

**Table 4:** Primary outcomes.

Primary outcomes^a,b^		Ex-ante criteria	Ex-post results
**Feasibility of the future main study**			
	Recruitment and randomization (villages)	70% of villages approached will agree to participate and accept randomization	100% (29/29 villages) agreed^b^
	Recruitment and randomization (households)	In participating villages, 70% of eligible households will agree to participate and accept randomization	98.0% (387/395 households contacted) agreed^b^
	Retention (households)	50% of households participating in the baseline survey will agree to participate in the end line survey	82.2% (318/387) enrolled households agreed^b^ and 2.1% (8/387) households refused
	Contamination	Contamination proportion between treatment groups should be <15%	0.6% (1/166 control end line respondents called); 0.07% (1/1310 unique callers to IVR system from a control village)^c^
**Uptake (adoption)**		50% of households recruited to the study will participate	94.0% (173/184) of households participated
	mHealth^d^ interventions	Either by listening to ≥1 mHealth item	67.4% (124/184) listened to ≥1 mHealth item
	Small group meetings	Or by attending ≥1 small group meeting	78.3% (144/184) attended ≥1 meeting

^a^Feasibility outcomes were computed using the intention-to-treat (ITT) sample of 387 households (184 IG and 203 CG). Uptake was computed using the ITT intervention group sample (184 households).

^b^See flow diagram (Figure 2).

^c^See Multimedia Appendix 1.

^d^mHealth: mobile health.

### Secondary Outcomes

#### Coverage

Uptake of the 3 intervention channels (mHealth vaccination reminders, mHealth edutainment capsules, and face-to-face small group meetings) differed among user segments (Tables 5 and 6 present modeled results; Supplementary Table 1 provides bivariate associations).

The ownership of a mobile phone was common among IG households (173/184, 94.0%) and a critical precondition for uptake of both mHealth strategies. Owing to the very few (n=11) households without a mobile phone and the prognostic importance of this variable, effect size estimates for mobile phone ownership are unreliable. However, estimates for other variables are, in principle, unbiased:

- mHealth audio vaccination reminders were accessed by 62.5% (115/184) of households. In addition to mobile phone ownership, 2 factors predicted higher uptake: high maternal education (secondary 9 years or higher vs none; OR 4.45, 95% CI 1.17-16.88; *P*=.03) and mothers’ ease of access to the mobile phone (OR 3.55, 95% CI 1.08-11.71;*P*=.04).
- mHealth edutainment capsules were accessed by 60.3% (111/184) of households. In addition to mobile phone ownership, intervention uptake was predicted by high (as compared with low) women’s empowerment (OR 3.29, 95% CI 1.28-8.47;*P*=.01), with some evidence of greater uptake by the lowest castes (members of scheduled castes vs general castes; OR 2.79, 95% CI 0.95-8.21;*P*=.06). However, poor phone network quality impeded the uptake of edutainment capsules (OR 0.29, 95% CI 0.12-0.71;*P*=.01).
- Face-to-face small group meetings were attended by 78.3% (144/184) of households. Living far from the meeting site reduced the uptake of small meetings (OR 0.07, 95% CI 0.02-0.33;*P* <.001); no other factor predicted uptake.

We also studied factors shaping the intensity of uptake. In modeled analyses, the number of mHealth items heard was influenced by 3 factors: mother’s possession of a mobile phone, mother’s ease of phone access, and women’s empowerment. The number of small group meetings attended was influenced by 2 factors: living far from the meeting site and women’s empowerment (Multimedia Appendix 1).

**Table 5:** Determinants of mobile health intervention uptake.

Variable^a,^^b,c,d^			Vaccination reminders				Edutainment		
			OR (95% CI)		*P* value		OR (95% CI)		*P* value
**Wealth quintile**									
	Poorest (Q1; reference)	—^e^				—		—	
	(Q2)	0.51 (0.12-2.15)		.36		0.42 (0.12-1.52)		.19	
	(Q3)	0.56 (0.21-1.51)		.26		0.78 (0.22-2.71)		.69	
	(Q4)	1.15 (0.36-3.64)		.83		0.74 (0.28-1.93)		.54	
	Highest (Q5)	0.43 (0.09-2.11)		.30		1.24 (0.40-3.92)		.71	
**Caste^d^**									
	General (reference)	—		—		—		—	
	Other backward caste	—		—		1.15 (0.36-3.67)		.81	
	Scheduled caste	—		—		2.79 (0.95-8.21)		.06	
**Education of mother**									
	None (reference)	—		—		—		—	
	Primary	0.80 (0.26-2.50)		.70		1.21 (0.53-2.79)		.65	
	Secondary or higher	4.45 (1.17-16.88)		.03		1.95 (0.56-6.80)		.29	
**Education of father**									
	None (reference)	—		—		—		—	
	Primary	2.01 (0.60-6.70)		.26		1.15 (0.45-2.94)		.77	
	Secondary or higher	2.01 (0.62-6.47)		.24		1.52 (0.52-4.44)		.45	
**HH^f^ owns phone**									
	Yes versus no	23.90 (5.09-112.1)		.001		16.80 (4.27-66.18)		.001	
**Cell network: poor**									
	Yes versus no	—		—		0.29 (0.12-0.71)		.01	
**HH phone belonging to mother**									
	Yes versus no	1.21 (0.5-2.61)		.64		—		—	
**Mother’s phone access**									
	Easy versus no access	3.55 (1.08-11.71)		.04		—		—	
**Mother can dial**									
	No versus yes	0.82 (0.27-2.55)		.74		—		—	
**Female empowerment**									
	Lowest (reference)	—		—		—		—	
	Average	—		—		0.96 (0.4-2.09)		.91	
	Highest	—		—		3.29 (1.28-8.47)		.01	

^a^Analyses based on the intention-to-treat intervention group sample comprising 184 households.

^b^Estimates produced using Firth logistic regression with cluster bootstrapped standard errors (1000 iterations).

^c^We present the full models implemented for each outcome. Potential determinants with no evidence of association at the *P*<.25 level were not included in the models.

^d^Caste categories from most to least advantaged: general, other backward caste, and scheduled caste. The scheduled tribe category is missing, as there are no tribes in the study area.

^e^—: empty cells signify that variables were not included in models. Please see the *Methods* section on *Multivariable Analyses* subheading Coverage for further details.

^f^HH: household.

**Table 6:** Determinants of face-to-face intervention uptake.

Variable^a,^^b,c,d^			Small group meetings		
			OR (95% CI)		*P* value
**Wealth quintile**					
	Poorest (Q1; reference)	—^e^		—	
	(Q2)	0.67 (0.12-3.69)		.64	
	(Q3)	0.60 (0.11-3.28)		.55	
	(Q4)	0.50 (0.08-3.00)		.45	
	Highest (Q5)	0.68 (0.16- 2.88)		.60	
**Education of mother**					
	None (reference)				
	Primary	0.62 (0.11-3.33)		.58	
	Secondary or higher	0.41 (0.04- 3.87)		.44	
**Education of father**					
	None (reference)	—		—	
	Primary	2.76 (0.32-23.70)		.35	
	Secondary or higher	2.84 (0.25-31.73)		.40	
**HH^d^ lives far**					
	No (reference)	—		—	
	Yes	0.07 (0.02-0.33)		.001	
**HH phone belonging to father**					
	Yes versus no	2.19 (0.6-8.03)		.24	
**HH phone belonging to someone else**					
	Yes versus no	0.41 (0.08-2.12)		.29	
**Mother can dial**					
	No versus yes	1.79 (0.2-15.63)		.60	
**Permission to attend meeting**					
	Yes versus no	0.69 (0.16-3.08)		.63	

^a^Analyses based on the intention-to-treat intervention group sample comprising 184 households.

^b^Estimates produced using the Firth logistic regression with cluster bootstrapped standard errors (1000 iterations).

^c^We present the full models implemented for each outcome. Potential determinants with no evidence of association at the *P*=.24 level were not included in the models.

^d^HH: household.

^e^—: empty cells signify that variables were not included in models.

#### Adequacy of the Program Theory

Immunization knowledge was low at baseline in both study groups. For 3 of the 4 indicators studied, knowledge improved in the IG at end line (Table 7). Differences-in-differences estimates of impact suggest that observed improvements were owing to the study interventions (Table 8). Effect sizes increased after adjustment for baseline imbalances.

For 8 of 9 intermediate outcomes, the regression results showed significantly higher basic health knowledge among the IG at end line (Table 9). For one topic (whether subjects had heard of diarrhea), knowledge at end line was equal for both treatment groups (*P*=.44). This was likely owing to an independent immunization and hygiene intervention in the study area run by the Gavi Alliance and Unilever.

**Table 7:** Proportion of correct responses on intermediate outcomes related to immunization knowledge, by study group.

Outcome^a^		Baseline			End line		
		Treated, n (%)	Control, n (%)	*P* value^b^	Treated, n (%)	Control, n (%)	*P* value^b^
**Knows immunization schedule^c^**							
	Yes	49 (26.6)	70 (34.5)	.095	102 (66.7)	74 (44.6)	<.001
**Knows how many times to vaccinate^d^**							
	Correct	2 (1.1)	3 (1.5)	.734	30 (19.6)	6 (3.6)	.001
“**On the vaccination card, what does each box represent?”^e^**							
	Correct	21 (11.4)	29 (14.3)	.400	42 (27.5)	34 (20.5)	.144
“**One should vaccinate a child with a minor illness”^f^**							
	True	112 (60.9)	119 (58.6)	.652	110 (71.9)	95 (57.2)	.006

^a^All responses are binary.

^b^*P* value for the chi-square test of independence.

^c^This is self-assessed knowledge of the schedule from birth to 5 years.

^d^The correct response is *7 times* before age 5.

eThe correct response is a vaccine dose.

^f^The correct response is *True*.

**Table 8:** Impact of the intervention on intermediate outcomes related to immunization knowledge.

Outcome^a^	Model 0^b^		Model 1^c^	
	OR (95% CI)	*P* value	OR (95% CI)	*P* value
Knows immunization schedule from birth to 5 years	7.87 (1.90-32.49)	.004	8.40 (2.05-34.35)	.003
Knows how many times to vaccinate by age 5	3.52 (2.08-5.98)	.001	4.21 (2.25-7.85)	.001
“On the vaccination card, what does each box represent?”	1.84 (1.12-3.03)	.016	2.00 (1.18-3.40)	.011
“Children with a minor illness should be vaccinated”	1.53 (0.72-3.28)	.27	1.54 (0.71-3.34)	.27

^a^These are differences-in-differences estimates of intervention impact.

^b^Model 0=unadjusted.

^c^Model 1=adjusted for variables imbalanced at the time of randomization (wealth index and cell network).

**Table 9:** Estimated probability of correct responses for intermediate outcomes reflecting basic health knowledge, intervention group versus controls.

Outcomes^a^			Model 0^b^				Model 1^c^		
			OR (95% CI)		*P* value		OR (95% CI)		*P* value
**Childhood pneumonia**									
	Has heard of	4.60 (2.68-7.89)		.001		4.98 (2.89-8.56)		.001	
	Can state signs	3.36 (1.58-7.13)		.002		3.67 (1.54-8.74)		.003	
	Can state how to prevent	4.12 (1.94-8.74)		.001		5.09 (2.16-12.02)		.001	
**Diarrhea**									
	Has heard of	1.24 (0.72-2.13)		.442		1.20 (0.74-1.96)		.456	
	Can state signs	4.14 (1.64-10.44)		.003		2.81 (1.48-5.32)		.002	
	Can state how to prevent	3.71 (2.06-6.67)		.001		3.82 (2.20-6.61)		.001	
**Dengue or chikungunya**									
	Has heard of	3.80 (2.35-6.10)		.001		3.97 (2.57-6.13)		.001	
	Can state how it is transmitted	3.61 (2.13-6.12)		.001		3.94 (2.45-6.31)		.001	
	Can state how to prevent	3.30 (1.97-5.53)		.001		3.53 (2.19-5.67)		.001	

^a^These are basic health topics other than immunization, evaluated only at study end line.

^b^Model 0=unadjusted.

^c^Model 1=adjusted for wealth index, maternal education, paternal education, caste, and women’s empowerment.

## Discussion

### Principal Findings

We conducted a pilot trial of SBCC interventions focusing on immunization and other basic themes important for child and family health. Interventions were delivered through small face-to-face meetings and via mobile phones using pushed audio messages and other strategies suitable for disadvantaged populations. The pilot trial aimed to assess the feasibility of a future planned main study and to draw lessons to optimize delivery of the interventions at scale.

This paper offers 4 salient findings: First, all ex-ante feasibility criteria related to recruitment, randomization, retention, and contamination were satisfied, providing compelling evidence that the planned future main trial is feasible as planned. Uptake of interventions (adoption) was near universal (50% ex-ante vs 94% in practice), demonstrating strong interest and acceptability. Second, analyses of uptake and use demonstrated that intervention use was shaped by social determinants but that the chosen combination of strategies reached all population groups, even the most vulnerable. Third, constellations of determinants differed by intervention delivery channel. Ownership of a mobile phone was critical for participation in mHealth (vaccination reminders and edutainment) interventions, whereas distance from place of residence to the meeting site was important for small group meetings. mHealth vaccination reminders were taken up preferentially by more educated women and those with easy phone access within the household, whereas mHealth edutainment capsules were favored by more empowered women and by lower caste groups, for whom the content was likely novel and useful. Face-to-face meetings were the most equitable intervention channel; participation was equal or higher among those with greater needs. Women’s empowerment was an important transversal determinant, increasing uptake and intensity for all interventions. Fourth, we found that the study interventions lead to measurable improvements in basic health knowledge, supporting the potential for impact at scale. Changes in intermediate outcomes are consistent with the intervention theory of change.

### Limitations

At least five important caveats should also be considered. First, an important potential bias relevant for the future definitive trial relates to vaccination coverage assessment. As documented in our pilot study and elsewhere, the population denominator used in administrative estimates is often inaccurate (and the reported number of doses unreliable) [27]. Although household surveys are considered superior, they are also affected by seasonal and chance variations and do not shed light directly on population immunity gaps. Improvements in vaccination coverage assessment methods would strengthen the ability of the main trial to assess immunization program performance. Second, those delivering and receiving interventions could not be blinded due to the nature of the interventions. We attempted to limit potential bias due to lack of blinding by hiring independent outcome assessors unaware of study purpose and group assignment, using indicators based on objective measures where possible, and triangulating between multiple measures and data sources to strengthen inference. Third, although interventions were able to achieve widespread reach in highly disadvantaged populations to ensure equity and impact, these findings are limited by the relatively small size of the IG. Fourth, quality of intervention delivery may be more difficult to achieve in a routine care setting. The personnel delivering the RCT interventions were highly motivated, well trained, and closely supervised. It may be difficult to maintain delivery quality at scale, particularly for face-to-face components. Fifth, the pilot study duration was too short (and sample size was too small) to assess definitive changes in health outcomes. Future research is required to demonstrate whether these gains in health knowledge result in improved vaccination coverage and child health.

### Generalizability

We highlight 3 insights relevant to the future definitive trial and other studies: The first relates to adapting mHealth interventions for highly disadvantaged populations. In rural India, mobile phone penetration and infrastructure is increasing rapidly, reducing barriers to delivering mHealth interventions. Our experience demonstrates that mHealth interventions can achieve reach and improve knowledge even in highly underprivileged populations, but that technical delivery and content must be substantially adapted. Although mHealth interventions using SMS have shown promise [6], we privileged audio messages with pushed dial outs owing to the low literacy, numeracy, and technological comfort levels of our target beneficiaries. We found that engaging story formats inclusive of diverse social groups were appreciated and that pure informational approaches such as vaccination reminders were taken up preferentially by the (relative) elite. As compared with SMS, audio messaging is more amenable to culture-specific contextualization and an edutainment approach. The second insight concerns how gender-related barriers shape immunization access [28] and affected the study interventions. Participation in mobile phone interventions was limited by women’s ease of access to mobile phones, and, to a lesser degree, with technological familiarity. Participation in face-to-face meetings was limited by norms governing women’s freedom of movement. Barriers were mitigated over time as families came to understand and value the interventions (Pérez et al, unpublished data, 2020). Unexpectedly, men, particularly fathers, were highly active participants. Future interventions should include a focus on men and families to strengthen inclusion and mitigate gender barriers. The third insight concerns the complementarity of mHealth and face-to-face communication: Although mHealth audio messaging is a promising strategy to deliver basic health information, our experience shows that it must be accompanied by face-to-face contact to enhance uptake [29] and equity. An mHealth strategy can extend the reach of face-to-face communication at high fidelity and low cost. Particularly among vulnerable populations, it is unlikely to fully replace in-person interaction. Future research exploring innovative delivery modalities while considering potential trade-offs between equity and efficiency (cost-effectiveness) is recommended.

### Conclusions

A novel SBCC intervention model using face-to-face and mHealth approaches is feasible to evaluate in a future randomized trial and has the potential to strengthen the delivery of immunization and universal primary health care. The interventions achieved widespread reach in a highly disadvantaged population and showed early evidence of impact on participants’ knowledge, supporting the intervention theory of change. Behavior change communication via mobile phones proved viable and contributed to standardization and scalability. Face-to-face interactions remain necessary to achieve equity and reach, suggesting the need for ongoing health system strengthening to accompany the introduction of promising mobile phone technologies.

## Appendix

Multimedia Appendix 1 Supplementary methods and results.

## Abbreviations

ANM: Assistant Nurse Midwives
ASHA: Accredited Social Health Activists
AWW: Anganwadi Workers
CG: control group
CTRI: Clinical Trials Registry–India
GoI: Government of India
GEE: generalized estimating equations
IG: intervention group
ITT: intention-to-treat
IVR: interactive voice response
LMIC: low- and middle-income country
mHealth: mobile health
RCT: randomized controlled trial
SBCC: social and behavior change communication
SDG: Sustainable Development Goals
UN: United Nations
UP: Uttar Pradesh
VHND: Village Health and Nutrition Day
WASH: water, sanitation, and hygiene
