# Supplementary material for: Social and Behavior Change Communication Interventions Delivered Face-to-Face and by a Mobile Phone to Strengthen Vaccination Uptake and Improve Child Health in Rural India: Randomized Pilot Study
Source: JMIR Mhealth Uhealth. 2020 Sep 21;8(9):e20356. doi: 10.2196/20356 (PMC7546625; doi:10.2196/20356)
Supplement: Multimedia Appendix 1 [file mhealth_v8i9e20356_app1.pdf]

Supplementary Appendix

Supplementary Methods

1.0 Detailed description of study interventions: TiDIER Checklist(Hoffmann et al., 2014) for the *Tika Vaani* Pilot

**Brief name**
**1 Provide the name or a phrase that describes the intervention**
Social and behaviour change communication for members of the general public in rural Indian villages addressing topics related to child health. The intervention combines edutainment and training capsules broadcast via mobile phone and community mobilisation activities.
**Why**
**2 Describe any rationale, theory, or goal of the elements essential to the intervention**
The Government of India offers Village Health and Nutrition Days (VHNDs) to make health services and information accessible to underserved rural communities. VHNDs are designed to provide first-contact primary health care to rural areas by bringing together a large package of basic health, nutrition, and sanitation services offered free-of-charge in a single location each month. Core services include those for reproductive, maternal, new-born and child health, including immunisation, tuberculosis and HIV treatment and control, and counselling for communicable disease prevention and health promotion.(Ministry of Health and Family Welfare Government of India, 2007) Despite improvements, India's under-5 mortality rate (48 deaths per 1,000 live births) remains high, health knowledge is low, poor health behaviours are common, and only 62% of children are fully immunized. Research shows that many essential services designed to be offered by VHNDs are currently not reaching the population effectively.(Johri et al., 2019) Building on the opportunity presented by widespread mobile phone ownership, we are piloting an interactive, voice-based, mobile platform (*Tika Vaani, or "vaccine voice"*) to respond to this gap, with a focus on child health. Tika Vaani's planned channels (1. general population 2. Community health workers (CHW)) will broadcast edutainment and training capsules and are combined with community mobilization activities. The primary objective of the main study is to increase immunization coverage among children 0-2 years old living in rural Hardoi district, Uttar Pradesh, India. The platform also includes content on additional themes important for children's health that are stipulated to be co-delivered with immunisation during VHNDs such as health education related to nutrition, and water, sanitation and hygiene (WASH). Services provided under the VHND umbrella are selected on the basis of their importance for population health based on India's burden of disease and scientific evidence of impact.(Ministry of Health and Family Welfare Government of India, 2007) Our intervention tests an alternative means to provide a subset of information relevant to child health deemed essential by VHNDs that is currently underprovided.(Johri et al., 2019) It thereby aims to support community health workers in their tasks, to empower families to better care for their children's health, and to enhance community engagement in health and health services.
**What**
**3 Materials: Describe any physical or informational materials used in the intervention, including those provided to participants or used in intervention delivery or in training of intervention providers. Provide information on where the materials can be accessed (such as online appendix, URL)**

The following materials will be used for intervention training and delivery.<br><br>• **Training of intervention providers**<br>To standardize the knowledge of the field staff, a manual containing key concepts for working with the community and a schedule of intervention activities will be used.<br>The manual is available from the principal investigator.<br><br>• **Delivery of the intervention**<br>a) Information delivery through <u>Introductory Meetings</u><br>A mobile phone and speaker will be used to disseminate information in the community.<br>We will promote the Tika Vaani intervention by painting the logo and telephone number on village walls using paint, a brush, and a stencil. Wall painting will be done at common visible places in the community after receiving permission from the relevant authorities. Pamphlets (sheets) and stickers containing the Tika Vaani phone number will also be distributed.<br>Other materials: Village map (drawn by field staff during the baseline survey), target households list, target households mobile number collection sheet, pamphlets and glue, community meeting introduction script and street play script<br><br>b) The intervention group will receive a series of information capsules delivered via mobile phone<br><br>An interactive voice response (IVR) system will be used where pre-recorded informative stories/capsules and messages will be available on different topics related to child health. Topic areas include: Village Health & Nutrition Days, immunisation, water storage, hand washing, diarrhoea management and prevention, pneumonia management and prevention, dengue and chikungunya management and prevention). Information capsules range in length from roughly 3 to 9 minutes, and reminder messages from 1 to 3 minutes.<br>Capsules are available from the principal investigator<br><br>c) Information delivered through <u>small group meetings</u><br>A structured meeting guide will be used to learn about the experiences and the perception of the community regarding the Tika Vaani Intervention. A standardized recording format will be used to gather information from participants attending small group meetings.<br>Small group meetings will be convened in quiet accessible places at different points in the village to enable broad participation based on village maps.
**4 Procedures: Describe each of the procedures, activities, and/or processes used in the intervention, including any enabling or support activities**
In each intervention group village, field team members will conduct a large introductory meeting to explain the intervention to the community. On the meeting day (but prior to the meeting), field team members will approach elected village leaders and frontline workers involved in VHND delivery (ASHA, Anganwadi, Sahaika, ANM if available) to discuss the intervention and seek their support, and to identify an appropriate meeting location. A place within the community accessible to all people will be selected; if all people cannot access the meeting, a single meeting will be held and target households (those with children in the age range of 0 to 12 months previously identified in the baseline survey) unable to participate will be visited personally following the meeting. Field team members will request permission to paint village walls to promote the intervention, and paint walls with the intervention name, logo, and phone number. The meeting will be announced throughout the village by going door-to-door, with a focus on target households with children in the age range of 0 to 12 months (previously identified in the baseline survey). Phone numbers will be collected in three stages: from target households during the baseline survey, from

target households on the day of the introductory meeting (to confirm or add numbers), and during the introductory meeting from attendees who dial the Tika Vaani number.

General Public

- Pushed Messages:
    - Over a 3-month period, 13 information capsules and 13 short reminder messages with content in basic areas of importance to child health (Village Health & Nutrition Days, immunisation, water storage, hand washing, diarrhoea management and prevention, pneumonia management and prevention, dengue and chikungunya management and prevention, will be "pushed" to families with young children in the age range of 0-12 months. One new information capsule and one summary capsule of key points will be pushed per week according to the schedule presented in Appendix A.
    - Timely reminder voice messages for immunisation will also be pushed based on the child's birthdate and the Government of India immunisation schedule. When a child's vaccination is due, families will receive three calls (four days prior, one day prior, and on the day of the VHND (vaccination day)) informing them that the child's vaccinations are due and to go to the local vaccination centre. Members of the general public in these villages are also eligible to receive pushed messages.
- On-demand access: Any person residing in an intervention village can phone the number to access content on demand through the IVR portal, to speak to an operator to facilitate linkage to content, or to leave a question or comment.
- Community meetings: In addition, a series of 4 community meetings (1 large group introductory meeting, 3 small group meetings held at monthly intervals) will be held. The introductory meeting will invite the entire village with the purpose of informing the community about the intervention and encouraging participation. Small group meetings will focus on families with children in the target age range, but both large and small meetings will be open to all. Each small group meeting will be replicated several times on a single day to enable widespread participation (for example, Small Group Meeting 1, conducted 4 times in Village 1 and 6 times in village 2). The number of small group meeting replications required will be established based on village size and knowledge of village social composition to enable widespread participation. Small group meetings will be held separately for men and women to optimize ease of communication. The primary focus will be on the child's primary caregiver (usually the mother) but other female family members and female members of the general public will be welcome to participate in women's meetings. Meetings with male family members and other male participants will be held separately.

Health workers

- Over the same 3-month period, health workers will receive early access to the same content and will be invited to contribute to leading small group meetings, with the support of intervention field staff. The goal is to build the capacity of health workers and to support them in their role.

All intervention components will be available free of charge to end users.

**Who provided**
**5 For each category of intervention provider (such as psychologist, nursing assistant), describe their expertise, background, and any specific training given**

The intervention will be implemented by paid field staff. We will endeavour to recruit equal numbers of males and females as experience shows that some activities are more successful if delivered by someone of the same gender. Selection criteria for field staff include an interest in promoting healthy lifestyles, residence in the intervention area (Hardoi district), completion of at least 12 years of schooling, experience

in working with the community, and ability to speak Hindi and the local dialect. Field staff will receive training on the *Tika Vaani* intervention to facilitate successful implementation. Competence in relevant skills will be assessed prior to the intervention. Ability to deliver the intervention will be assessed on an ongoing basis by field supervisors; those deemed weak in specific areas will be retrained.

A recognised social marketing and technology company designed and will implement the IVR system. The company was chosen for its experience in using mobile technology to improve the quality of life of rural Indian communities. (For more information, please consult https://gramvaani.org/)

Frontline workers involved in delivery of Village Health and Nutrition Days (ASHA, Anganwadi worker, ANM) are not direct intervention providers, but they will be invited to participate to encourage use of the *Tika Vaani* platform and to support community discussions. These workers are women selected and trained by the Government of India to work as an interface between the community and the public health and social welfare systems of India. The participation requested falls under the scope of their ordinary responsibilities and no additional remuneration or incentives will be provided.

How

6 Describe the modes of delivery (such as face to face or by some other mechanism, such as internet or telephone) of the intervention and whether it was provided individually or in a group

The intervention will be delivered in two ways:

1. Community meetings delivered face-to-face in groups.
   Within each intervention village, two types of meetings will be organized: 1) a large meeting with the entire community to introduce the intervention and 2) meetings in smaller groups to discuss content themes. Meetings will be initiated by field team members according to the intervention plan (1 large group introductory meeting, 3 small group meetings held at monthly intervals). Meeting size is variable. The large meeting size depends on the size of the community and typically ranges from roughly 50 to 200. Small meetings typically range from 5 to 25 participants. All meetings are interactive.
2. Information / edutainment capsules and immunisation reminders delivered by mobile phone
   Information capsules will be delivered through the IVR system to listeners at their convenience. People may listen individually but are encouraged to listen and discuss as a family either through use of a speaker phone functionality or by calling the *Tika Vaani* number. Information capsules will be pushed at regular intervals by the system, and will also be accessible on demand.

Where

7 Describe the type(s) of location(s) where the intervention occurred, including any necessary infrastructure or relevant features

The intervention will be carried out in rural villages of Hardoi district, Uttar Pradesh. Community meetings will be conducted at convenient gathering points within villages. Those who listen via mobile phone will do so at their convenience, generally in their own homes.

When and How Much

8 Describe the number of times the intervention was delivered and over what period of time including the number of sessions, their schedule, and their duration, intensity, or dose

The intervention will be offered over a three-month period according to the schedule presented in Appendix A. Briefly:

**Delivery of the intervention**<br>a) Introductory Meeting<br>This meeting will be held in each village with the whole community once at the beginning of the intervention with duration of around 30-45 minutes. These meetings will be usually held in the day.<br>b) Information capsules delivered through IVR system<br>At the beginning of each week, families will receive a call with a specific health information capsule. Five days later, they will receive a call with the corresponding reminder capsule. Twelve information capsules and twelve reminder capsules will be released over the three months of the intervention. Information capsules ranged in length from roughly 3 to 9 minutes, and reminder messages from 1 to 3 minutes. During the week, participants can contact the IVR system at any time to repeat the information or to obtain other information of interest.<br>c) Small group meetings<br>A sequence of 4 community meetings (1 large group introductory meeting, 3 small group meetings held at monthly intervals) will be held in each intervention village. The introductory meeting will invite the entire village with the purpose of informing the community about the intervention and encouraging participation. Small group meetings will focus on families with children in the target age range, but both large and small meetings will be open to all. Each meeting in the series of 3 small group meetings will be replicated several times per village to enable widespread participation. The number of replicates will be established based on village size and knowledge of village social composition. Small group meetings will be held separately for men and women to optimize ease of communication. Meetings will be held every four weeks. Each meeting will last around one hour and will be held at a time and place that is convenient for the participants.
**Tailoring**
**9 If the intervention was planned to be personalised, titrated or adapted, then describe what, why, when, and how**
All participants will receive an identical intervention; there is no plan for tailoring.
**Modifications**
**10* If the intervention was modified during the course of the study, describe the changes (what, why, when, and how)**
The intervention was not modified; see the report on fidelity assessment.
**How well**
**11 Planned: If intervention adherence or fidelity was assessed, describe how and by whom, and if any strategies were used to maintain or improve fidelity, describe them**
To evaluate the fidelity of the intervention, observations will be carried out with a checklist in order to evaluate if the activities were carried out as planned in terms of coverage, frequency and duration and to report any modifications during the implementation of the intervention. Complementary qualitative research will also be conducted.
**12* Actual: If intervention adherence or fidelity was assessed, describe the extent to which the intervention was delivered as planned**<br><br>The intervention fidelity assessment will be published separately.

Additional: Services received by the control group. The control group received all standard health and welfare services offered by the Government of India. Most important among these are Village Health and Nutrition Days (VHNDs), which are designed to provide first-contact primary health care to rural areas by bringing together a large package of basic health, nutrition, and sanitation services offered free-of-charge in a single location each month. Core services include those for reproductive, maternal, new-born and child health, including immunisation, tuberculosis and HIV treatment and control, and counselling for communicable disease prevention and health promotion.(Ministry of Health and Family Welfare Government of India, 2007) Other programmes offered in the study area and designed to work synergistically with VHNDs include India's Integrated Child Development Services (ICDS) scheme, which offers nationwide nutrition and health promotion(Integrated Child Development Scheme (ICDS)), the Janani Suraksha Yojana (JSY), a conditional cash transfer program launched in 2005 to reduce maternal and neonatal mortality by increasing births in health facilities(Ministry of Health and Family Welfare Government of India, 2013), and Mission Indradhanush (MI), launched in 2014 (and intensified in 2017(Gurnani et al., 2018)) to close existing vaccination coverage gaps through a focus on lagging areas and populations to ensure that all children will benefit from vaccination against seven vaccine preventable diseases by 2020.(Ministry of Health and Family Welfare Government of India, 2015)

Appendix A

Month	Week	Capsule name<br>5 days main capsule + 2 days reminder capsule =7 days
**19th Jan – 19th Feb**	**Baseline survey (Water Storage)**	
**21st March to 31st March**	**Introductory meetings (big meetings)**	
2nd Apr – 6th Apr	1st week	Story: Pneumonia Management part-1
7th Apr – 8th Apr		Reminder capsule: Pneumonia Management part-1
9th Apr – 13th Apr	2nd week	Story: Pneumonia prevention part-2
14th Apr – 15th Apr		Reminder capsule: Pneumonia prevention part-2
16th Apr – 20th Apr	3rd week	Story: VHND
21st Apr – 22nd Apr		Reminder capsule: VHND
23rd Apr – 27th Apr	4th week	Story: FAQ2
28th Apr – 29th Apr		Reminder capsule: FAQ 2
**Discussion with small groups in the villages about played capsule in past 4 weeks**		
**2nd May to 14th May**	**First small group meetings**	
30th Apr - 4th May	5th week	Story: Malti Ki Koshish
5th May – 6th May		Capsule reminder: Malti Ki Koshish
7th May – 11th May	6th week	Story: Teeke Muft Bacche Chust
12th May – 13th May		Reminder capsule: Teeke Muft Bacche Chust
14th May – 18th May	7th week	Story: Samajhdar Saheli
19th May – 20th May		Reminder capsule: Samajhdar Saheli
21st May – 25th May	8th week	Story: Teekakaran Ki Kimat
26th May – 27th May		Reminder capsule: Teekakaran Ki Kimat
**Discussion with small groups in the villages about played capsule in past 4 weeks**		
**8th June to 20th June**	**Second small group meetings**	
28th May – 1st June	9th week	Story: Diarrhoea Management
2nd June – 3rd June		Reminder capsule: Diarrhoea Management
4th June – 8th June	10th week	Story: Diarrhoea Prevention
9th June – 10th June		Reminder capsule: Diarrhoea Prevention
11th June – 15th June	11th week	Story: Germ Concept
16th June – 17th June		Reminder capsule: Germ Concept
18th June – 22nd June	12th week	Story: Dengue and Chikungunya Part-1
23rd June – 24th June		Reminder capsule: Dengue and Chikungunya Part-1
26th June – 30th June	IVR survey of eligible household by TV team	
25th June – 29th June	13th week	Story: Dengue and Chikungunya Part-2
30th June – 1st July		Reminder capsule: Dengue and Chikungunya Part-2
2nd July – 9th July	Third small group meetings	
5th July – 7th August	IVR survey (Target households and general public) by Oniondev	
17 July – 20th August	Endline survey (Capsule-Handwashing)	
18th July – 6th September	Exit interview	

2.0 Assessment of Contamination

We assessed contamination using two data sources: (1) the IVR platform; (2) the household end line survey.

1. IVR Platform

We examined all calls received during the study intervention period from March 21st, 2018 (the date when promotion of the Tika Vaani number was initiated in the 13 pilot study intervention villages) to August 20th, 2018 (the last day of the end line survey in the 26 pilot study villages). There were 1310 unique mobile numbers from which we received calls from during the study dates. After removing 180 mobile numbers pre-registered in our database (as belonging to target families or frontline workers) and 651 mobile numbers that occurred contemporaneously with meetings held in intervention group villages (when people were encouraged to dial the number to be registered in the database), we identified 479 unknown calls representing candidates for contamination. Field staff contacted these numbers to verify their origin.

We found that 162/479 (33.8%) calls belonged to members of the intervention group, of which 16/479 (3.3%) belonged to target households and 146/479 (30.5%) belonged to the general population. Only 1/479 (0.2%) originated in the control group; the call was from a family belonging to the general population. We identified 96/479 calls (20%) that originated from outside of the 26 villages involved in the pilot intervention. A further 220/479 (45.9%) of mobile numbers were unable to be reached due to being switched off, out of reach, not in service, or unanswered.

Over the 5-month study intervention period, contamination from control villages was extremely low. There were 0 calls received from the control village target population (0/1310; 0%), and 1 call received from the control village general population (1/1310; 0.08%). There were 97 documented calls from non-study villages (96 calls from non-study villages + 1 control village call)/1310 (7.4%) and a total of 317 calls possibly originating from outside pilot study intervention villages (96 calls from non-study villages + 1 control village call + 220 unreachable)/1310 (24.2%).

Although the main study duration will be longer than that of the pilot study, we conclude that contamination from control villages is unlikely to have an important impact on the validity of the main study findings (for which the main comparison of interest is the contrast between intervention and control group results). Additional information may help to clarify our interpretation. Prior to launching the pilot study, we engaged in extensive pilot testing from January 1st, 2017 through January 10th, 2018 in more than 38 other villages and the same mobile number was circulated in some of these villages. Of the 96 calls originating from outside of the 26 pilot study villages whose origins could be traced, calls from previously contacted areas represented a vast majority.

**Calls received from unique mobile numbers: period from 21st March to 20th August**				
Call details	General population	Target Household	Frontline workers	Total
From intervention villages	801	164	28	993
From control villages	1	0	0	1
From non-study villages	-	-	-	96

Not in existing data/Phone switched off/out of reach/disconnected/ not receive/wrong no	-	-	-	220
**Total**	**-**	-	-	1310

2. Household end line survey

The end line survey administered to all (intervention group (IG) and control group (CG)) households included relevant assessment questions.

- Have you heard of Tika Vaani? (77% (118/153) IG versus 1% (2/166) CG)

- Have you or your family members ever used Tika Vaani? (69% (106/153) IG versus 1% (1/166) CG)

These responses corroborated the information that contamination from control villages was extremely low.

References

GURNANI, V., HALDAR, P., AGGARWAL, M. K., DAS, M. K., CHAUHAN, A., MURRAY, J., ARORA, N. K., JHALANI, M. & SUDAN, P. 2018. Improving vaccination coverage in India: lessons from Intensified Mission Indradhanush, a cross-sectoral systems strengthening strategy. *BMJ,* 363**,** k4782.

HOFFMANN, T. C., GLASZIOU, P. P., BOUTRON, I., MILNE, R., PERERA, R., MOHER, D., ALTMAN, D. G., BARBOUR, V., MACDONALD, H., JOHNSTON, M., LAMB, S. E., DIXON-WOODS, M., MCCULLOCH, P., WYATT, J. C., CHAN, A. W. & MICHIE, S. 2014. Better reporting of interventions: template for intervention description and replication (TIDieR) checklist and guide. *BMJ,* 348**,** g1687.

INTEGRATED CHILD DEVELOPMENT SCHEME (ICDS). Available: http://icds-wcd.nic.in/icds.aspx [Accessed April 6 2018].

JOHRI, M., RODGERS, L., CHANDRA, D., ABOU RIZK, C., NASH, E. & MATHUR, A. K. 2019. Implementation fidelity of Village Health and Nutrition Days in Hardoi District, Uttar Pradesh, India: A cross-sectional survey.

MINISTRY OF HEALTH AND FAMILY WELFARE GOVERNMENT OF INDIA 2007. Monthly Village Health Nutrition Day: Guidelines for AWWs/ ASHAs/ ANMs/ PRIs.

MINISTRY OF HEALTH AND FAMILY WELFARE GOVERNMENT OF INDIA. 2013. *NRHM Componnets : Reproductive & Child Health : JSY* [Online]. Available: http://nhm.gov.in/nrhmcomponnets/reproductive-child-health/jsy.html [Accessed December 14, 2017 2017].

MINISTRY OF HEALTH AND FAMILY WELFARE GOVERNMENT OF INDIA 2015. Mission Indradhanush - Operational Guidelines. New Delhi.

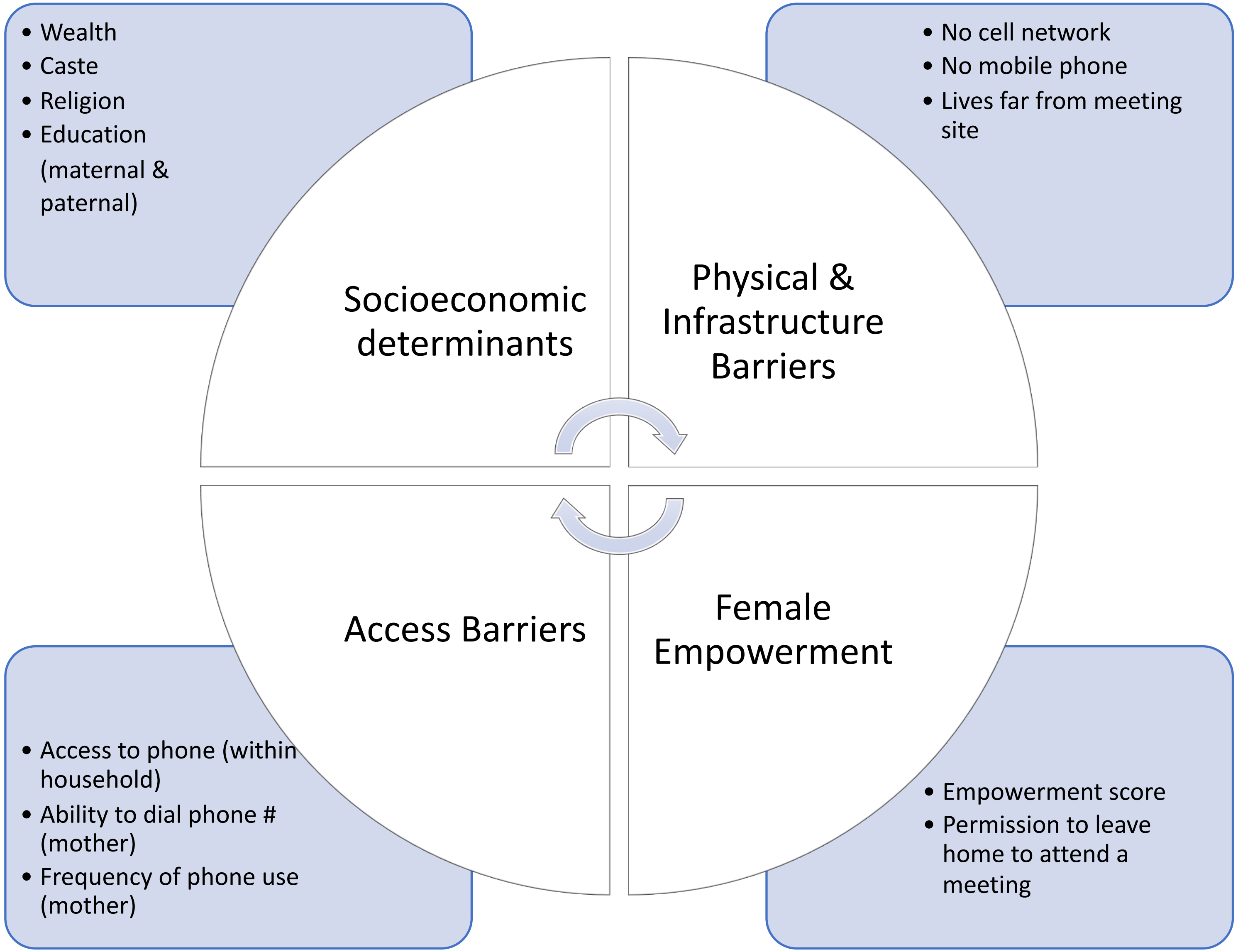

Supplementary Figure 1: Conceptual framework for the statistical analysis of uptake and coverage

Supplementary Table 1: Bivariate associations between predictors of participation and uptake of interventions for 184 intervention group households

	mHealth: Vaccination reminders			mHealth: Edutainment capsules			Face-to-Face: Small group meetings		
	No, n(%)	Yes; n(%)	p-value	No, n(%)	Yes; n(%)	p-value	No, n(%)	Yes; n(%)	p-value
Proportion of	69(37.5)	115(62.5)		111(60.33)	73(39.67)		40(21.74)	144(78.26)	
Wealth Index (quintile)									
Lowest (Q1)	19(39.58)	29(60.42)		27(56.25)	21(43.75)		8(16.67)	40(83.33)	
(Q2)	20(54.05)	17(45.95)		29(78.38)	8(21.62)		4(10.81)	33(89.19)	
(Q3)	14(37.84)	23(62.16)	0.052	22(59.46)	15(40.54)	0.153	8(21.62)	29(78.38)	0.118
(Q4)	5(17.86)	23(82.14)		15(53.57)	13(46.43)		9(32.14)	19(67.86)	
Highest (Q5)	11(32.35)	23(67.65)		18(52.94)	16(47.06)		11(32.35)	23(67.65)	
Religion[2]									
Hindu	69(38.12)	112(61.88)		110(60.77)	71(39.23)		39(21.55)	142(78.45)	
Muslim	0(0.00)	3(100.00)	0.176	1(33.33)	2(66.67)	0.335	1(33.33)	2(66.67)	0.624
Caste[3]									
General	12(31.58)	26(68.42)		23(60.53)	15(39.47)		11(28.95)	27(71.05)	
OBC	35(39.33)	54(60.67)	0.696	61(68.54)	28(31.46)	0.039	17(19.10)	72(80.90)	0.463
SC	22(38.60)	35(61.40)		27(47.37)	30(52.63)		12(21.05)	45(78.95)	
Maternal education (years)									
None (0)	30(48.39)	32(51.61)		43(69.35)	19(30.65)		8(12.90)	54(87.10)	
Primary (1 to 8)	36(42.35)	49(57.65)	p<0.001	49(57.65)	36(42.35)	0.164	20(23.53)	65(76.47)	0.064
Secondary (9 to 12), or higher	3(8.11)	34(91.89)		19(51.35)	18(48.65)		12(32.43)	25(67.57)	
Paternal education (years)									
None (0)	19(65.52)	10(34.48)		23(79.31)	6(20.69)		5(17.24)	24(82.76)	
Primary (1 to 8)	32(35.56)	58(64.44)	0.002	54(60.00)	36(40.00)	0.047	16(17.78)	74(82.22)	0.190
Secondary (9 to 12), or higher	18(27.69)	47(72.31)		34(52.31)	31(47.69)		19(29.23)	46(70.77)	
Household lives far from meeting site[4]									
No	54(38.03)	88(61.97)		89(62.68)	53(37.32)		15(10.56)	127(89.44)	
Yes	15(35.71)	27(64.29)	0.786	22(52.38)	20(47.62)	0.231	25(59.52)	17(40.48)	p<0.001
Cell phone network quality - poor									
No	61(37.42)	102(62.58)		93(57.06)	70(42.94)		37(22.70)	126(77.30)	
Yes	8(38.10)	13(61.90)	0.952	18(85.71)	3(14.29)	0.012	3(14.29)	18(85.71)	0.379
Household owns a mobile phone									
No	11(100.00)	0(0.00)	p<0.001	11(100.00)	0(0.00)	0.006	2(18.18)	9(81.82)	0.768
Yes	58(33.53)	115(66.47)		100(57.80)	73(42.20)		38(21.97)	135(78.03)	
In this household, mother has a phone									
No	45(42.45)	61(57.55)		64(60.38)	42(39.62)		21(19.81)	85(80.19)	
Yes	24(30.77)	54(69.23)	0.106	47(60.26)	31(39.74)	0.987	19(24.36)	59(75.64)	0.460
In this household, father has a phone									

	mHealth: Vaccination reminders			mHealth: Edutainment capsules			Face-to-Face: Small group meetings		
	No, n(%)	Yes; n(%)	p-value	No, n(%)	Yes; n(%)	p-value	No, n(%)	Yes; n(%)	p-value
No	28(41.18)	40(58.82)		40(58.82)	28(41.18)		18(26.47)	50(73.53)	
Yes	41(35.34)	75(64.66)	0.430	71(61.21)	45(38.79)	0.750	22(18.97)	94(81.03)	0.234
In this household, someone else has a phone									
No	66(40.74)	96(59.26)		100(61.73)	62(38.27)		32(19.75)	130(80.25)	
Yes	3(13.64)	19(86.36)	0.014	11(50.00)	11(50.00)	0.291	8(36.36)	14(63.64)	0.076
Mother's mobile phone access									
Cannot access	35(38.89)	55(61.11)		58(64.44)	32(35.56)		21(23.33)	69(76.67)	
Can use easily	34(36.17)	60(63.83)	0.703	53(56.38)	41(43.62)	0.264	19(20.21)	75(79.79)	0.608
Mother can dial a phone number?									
Yes	44(32.12)	93(67.88)		76(55.47)	61(44.53)		35(25.55)	102(74.45)	
No	25(53.19)	22(46.81)	0.010	35(74.47)	12(25.53)	0.020	5(10.64)	42(89.36)	0.032
Mother’s frequency of mobile phone use									
Rarely	11(40.74)	16(59.26)		15(55.56)	12(44.44)		4(14.81)	23(85.19)	
When needed	40(39.22)	62(60.78)		63(61.76)	39(38.24)	0.841	21(20.59)	81(79.41)	0.400
Almost daily	18(32.73)	37(67.27)	0.676	33(60.00)	22(40.00)		15(27.27)	40(72.73)	
Women’s empowerment score (tercile)									
Lowest	28(33.73)	55(66.27)		52(62.65)	31(37.35)		20(24.10)	63(75.90)	
Average	32(43.24)	42(56.76)	0.418	48(64.86)	26(35.14)	0.076	15(20.27)	59(79.73)	0.767
Highest	9(33.33)	18(66.67)		11(40.74)	16(59.26)		5(18.52)	22(81.48)	
Permission to attend a meeting in the village									
No	42(37.84)	69(62.16)		67(60.36)	44(39.64)		19(17.12)	92(82.88)	
Yes	27(36.99)	46(63.01)	0.907	44(60.27)	29(39.73)	0.991	21(28.77)	52(71.23)	0.061

[1] This is the p-value for the χ^2 test of independence

[2] This is the religion of the household head.

[3] Caste was categorized as for Indian national surveys. “General” is the most advantaged category, followed by “OBC” (other backward caste), and “SC” (scheduled caste). The category “ST” (scheduled tribe) is missing as there are no tribes in the study area. By convention, caste categories are applied to all population groups, irrespective of religion. For two households, missing data on caste were imputed based on locality of residence within the village. There were no other missing data.

[4] For intervention group households only, implementation teams assessed whether or not households were able to attend small group meetings based on geographical distance from their place of residence to anticipated meeting sites. This categorization was made at study baseline, prior to undertaking interventions.

Supplementary Table 2: Bivariate associations characterising intensity of participation in interventions for 184 intervention group households

	mHealth: # of items heard				Face-to-Face: # of Small group meetings			
	None n(%)	1 to 7 n(%)	≥ 8 n(%)	p-value	None n(%)	One n(%)	2 or 3 n(%)	p-value
Proportion of	61(33.15)	62(33.70)	61(33.15)		40(21.74)	70(38.04)	74(40.22)	
Wealth Index (quintile)								
Lowest (Q1)	17(35.42)	12(25.00)	19(39.58)		8(16.67)	19(39.58)	21(43.75)	
(Q2)	20(54.05)	9(24.32)	8(21.62)		4(10.81)	13(35.14)	20(54.05)	
(Q3)	11(29.73)	15(40.54)	11(29.73)	0.072	8(21.62)	14(37.84)	15(40.54)	0.254
(Q4)	5(17.86)	12(42.86)	11(39.29)		9(32.14)	12(42.86)	7(25.00)	
Highest (Q5)	8(23.53)	14(41.18)	12(35.29)		11(32.35)	12(35.29)	11(32.35)	
Religion[2]								
Hindu	61(33.70)	60(33.15)	60(33.15)		39(21.55)	69(38.12)	73(40.33)	
Muslim	0(0.00)	2(66.67)	1(33.33)	0.369	1(33.33)	1(33.33)	1(33.33)	0.886
Caste[3]								
General	9(23.68)	17(44.74)	12(31.58)		11(28.95)	16(42.11)	11(28.95)	
OBC	31(34.83)	34(38.20)	24(26.97)	0.044	17(19.10)	30(33.71)	42(47.19)	0.343
SC	21(36.84)	11(19.30)	25(43.86)		12(21.05)	24(42.11)	21(36.84)	
Maternal education (years)								
None (0)	27(43.55)	18(29.03)	17(27.42)		8(12.90)	24(38.71)	30(48.39)	
Primary (1 to 8)	32(37.65)	23(27.06)	30(35.29)	p<0.001	20(23.53)	34(40.00)	31(36.47)	0.181
Secondary (9 to 12), or higher	2(5.41)	21(56.76)	14(37.84)		12(32.43)	12(32.43)	13(35.14)	
Paternal education (years)								
None (0)	17(58.62)	7(24.14)	5(17.24)		5(17.24)	12(41.38)	12(41.38)	
Primary (1 to 8)	29(32.22)	33(36.67)	28(31.11)	0.011	16(17.78)	31(34.44)	43(47.78)	0.169
Secondary (9 to 12), or higher	15(23.08)	22(33.85)	28(43.08)		19(29.23)	27(41.54)	19(29.23)	
Household lives far from meeting site[4]								
No	49(34.51)	47(33.10)	46(32.39)		15(10.56)	55(38.73)	72(50.70)	
Yes	12(28.57)	15(35.71)	15(35.71)	0.772	25(59.52)	15(35.71)	2(4.76)	p<0.001
Cell phone network quality - poor								
No	53(32.52)	53(32.52)	57(34.97)		37(22.70)	63(38.65)	63(38.65)	
Yes	8(38.10)	9(42.86)	4(19.05)	0.335	3(14.29)	7(33.33)	11(52.38)	0.446
Household owns a mobile phone								
No	11(100.00)	0(0.00)	0(0.00)		2(18.18)	5(45.45)	4(36.36)	
Yes	50(28.90)	62(35.84)	61(35.26)	p<0.001	38(21.97)	65(37.57)	70(40.46)	0.869
In this household, mother has a phone								
No	39(36.79)	36(33.96)	31(29.25)		21(19.81)	43(40.57)	42(39.62)	
Yes	22(28.21)	26(33.33)	30(38.46)	0.340	19(24.36)	27(34.62)	32(41.03)	0.648
In this household, father has a phone								
No	27(39.71)	16(23.53)	25(36.76)		18(26.47)	23(33.82)	27(39.71)	
Yes	34(29.31)	46(39.66)	36(31.03)	0.077	22(18.97)	47(40.52)	47(40.52)	0.444
In this household, someone else has a phone								
No	58(35.80)	52(32.10)	52(32.10)		32(19.75)	61(37.65)	69(42.59)	
Yes	3(13.64)	10(45.45)	9(40.91)	0.114	8(36.36)	9(40.91)	5(22.73)	0.110
Mother's mobile phone access								
Cannot access	33(36.67)	26(28.89)	31(34.44)		21(23.33)	33(36.67)	36(40.00)	
Can use easily	28(29.79)	36(38.30)	30(31.91)	0.377	19(20.21)	37(39.36)	38(40.43)	0.863
Mother can dial a phone number?								
Yes	37(27.01)	51(37.23)	49(35.77)		35(25.55)	47(34.31)	55(40.15)	
No	24(51.06)	11(23.40)	12(25.53)	0.010	5(10.64)	23(48.94)	19(40.43)	0.062
Mother's frequency of mobile phone use								
Rarely	10(37.04)	5(18.52)	12(44.44)		4(14.81)	11(40.74)	12(44.44)	
When needed	36(35.29)	34(33.33)	32(31.37)	0.289	21(20.59)	39(38.24)	42(41.18)	0.759
Almost daily	15(27.27)	23(41.82)	17(30.91)		15(27.27)	20(36.36)	20(36.36)	
Women's empowerment score (tercile)								
Lowest	24(28.92)	32(38.55)	27(32.53)		20(24.10)	35(42.17)	28(33.73)	
Average	30(40.54)	24(32.43)	20(27.03)	0.114	15(20.27)	22(29.73)	37(50.00)	0.215
Highest	7(25.93)	6(22.22)	14(51.85)		5(18.52)	13(48.15)	9(33.33)	
Permission to attend a meeting in the village								
No	37(33.33)	34(30.63)	40(36.04)		19(17.12)	42(37.84)	50(45.05)	
Yes	24(32.88)	28(38.36)	21(28.77)	0.476	21(28.77)	28(38.36)	24(32.88)	0.112

[1] This is the p-value for the χ^2 test of independence

[2] This is the religion of the household head.

[3] Caste was categorized as for Indian national surveys. "General" is the most advantaged category, followed by "OBC" (other backward caste), and "SC" (scheduled caste). The category "ST" (scheduled tribe) is missing as there are no tribes in the study area. By convention, caste categories are applied to all population groups, irrespective of religion. For two households, missing data on caste were imputed based on locality of residence within the village. There were no other missing data.

[4] For intervention group households only, implementation teams assessed whether or not households were able to attend small group meetings based on geographical distance from their place of residence to anticipated meeting sites. This categorization was made at study baseline, prior to undertaking interventions.

Supplementary Table 3: Determinants of intensity of participation in interventions[1, 2]

	mHealth: # items heard			Face-to-Face: # Small group meetings[3]		
	**OR**	**95%CI**	**P-value**	**OR**	**95%CI**	**P-value**
Wealth Index (quintile)						
(Q2) vs Lowest (Q1)	0.48	(0.16 - 1.39)	0.173	0.62	(0.23 - 1.65)	0.335
(Q3) vs (Q1)	0.56	(0.21 - 1.47)	0.239	0.53	(0.21 - 1.34)	0.181
(Q4) vs (Q1)	0.68	(0.25 - 1.90)	0.466	0.37	(0.14 – 1.00)	0.051
Highest (Q5) vs (Q1)	0.64	(0.18 - 2.27)	0.486	0.66	(0.21 - 2.07)	0.472
Caste[3]						
OBC vs General	0.98	(0.40 - 2.36)	0.959	1.35	(0.61 – 3.00)	0.462
SC vs General	1.71	(0.59 - 4.97)	0.327	1.35	(0.56 - 3.24)	0.500
Maternal education (years)						
Primary (1 to 8) vs None (0)	0.80	(0.32 - 1.99)	0.629	0.52	(0.22 - 1.22)	0.135
Secondary (9 to 12+) vs None (0)	1.58	(0.61 - 4.10)	0.349	0.43	(0.15 - 1.18)	0.101
Paternal education (years)						
Primary (1 to 8) vs None (0)	1.52	(0.51 - 4.50)	0.450			
Secondary (9 to 12+) vs None (0)	2.42	(0.71 - 8.23)	0.157			
Household lives far from meeting site[4]				0.07	(0.03 - 0.16)	p<0.001
Yes vs No	0.75	(0.30 - 1.88)	0.544			
Household owns a mobile phone						
Yes vs No	0.85	(0.40 - 1.80)	0.663			
Cell phone network quality - poor				1.66	(0.60 - 4.59)	0.327
Yes vs No						
In this household, mother has a phone				1.62	(0.45 - 5.92)	0.462
Yes vs No	25.99	(1.71 - 395.63)	0.019			
Mother's mobile phone access						
Can use easily vs Cannot access	24.28	(1.72 - 342.96)	0.018	1.82	(0.48 - 6.85)	0.375
Mother can dial a phone number						
No vs Yes	0.55	(0.21 - 1.44)	0.222	0.67	(0.29 - 1.54)	0.344
Women’s empowerment score (tercile)						
Average vs Lowest	0.70	(0.34 - 1.41)	0.313	2.34	(1.18 - 4.63)	0.015
Highest vs Lowest	2.78	(1.03 - 7.48)	0.043	1.24	(0.51 - 2.99)	0.637

1. These are ordered logistic regression models with robust standard errors
2. Tables present the full models implemented for each outcome. Potential determinants described in our conceptual framework that showed no evidence of association at the p<0.25 level with any outcome were not included in models.
3. Caste was categorized as for Indian national surveys. “General” is the most advantaged category, followed by “OBC” (other backward caste), and “SC” (scheduled caste). The category “ST” (scheduled tribe) is missing as there are no tribes in the study area. By convention, caste categories are applied to

all population groups, irrespective of religion. For two households, missing data on caste were imputed based on locality of residence within the village. There were no other missing data.

4. For intervention group households only, implementation teams assessed whether or not households were able to attend small group meetings based on geographical distance from their place of residence to anticipated meeting sites. This categorization was made at study baseline, prior to undertaking interventions.
